# The progress in the relationship between trace elements and acute lymphoblastic leukemia

**DOI:** 10.3389/fcell.2023.1145563

**Published:** 2023-03-09

**Authors:** Jing Wang, Pei Huang, Changhui Lang, Yan Luo, Zhixu He, Yan Chen

**Affiliations:** ^1^ Department of Pediatrics, Affiliated Hospital of Zunyi Medical University, Zunyi, China; ^2^ Department of Pediatrics, Guizhou Children’s Hospital, Zunyi, China; ^3^ Collaborative Innovation Center for Tissue Injury Repair and Regenerative Medicine of Zunyi Medical University, Zunyi, China

**Keywords:** trace elements, acute lymphoblastic leukemia, DNA synthesis, free radical, gene mutation

## Abstract

Trace elements are very important substances with low content in the human body. If the content of some trace elements changes, they are also related to diseases. Acute lymphoblastic leukemia (ALL) is a malignant blood tumor, and its relationship with trace elements has also been a concern by scholars. Not only have the trace element levels in ALL patients changed, but the efficacy of different treatment methods has also been linked to the corresponding trace element changes. The characteristics of ALL may be related to the dysregulation of differentiation and proliferation of lymphoid precursor cells. Cell proliferation and differentiation are often affected by changes in DNA levels. However, trace elements are involved in DNA damage and repair mechanisms. In recent years, as an increasing number of studies believe that ALL is related to the abnormal metabolism of trace elements in the body, this paper intends to discuss the research progress on the relationship between trace elements and ALL to provide more information on trace elements for the diagnosis, treatment, and prevention of ALL.

## Introduction

As the most common childhood cancer, acute lymphoblastic leukemia (ALL) accounts for 26% of tumors in children under 15% and 8% in adolescents between 15–19 years of age (Ward et al., 2014). ALL is an aggressive and complex disease, and the epidemiological survey data showed that its age-standardized incidence is 0.68/1000,000 (Yi et al., 2020; Genescà and González-Gil, 2022). ALL stands for T/B precursor lymphocyte malignancy and blocks lymphocyte differentiation, allowing cells to proliferate and survive abnormally (Zuckerman and Rowe, 2014). The possible pathogenesis of ALL contains redox imbalance (Chen et al., 2021), metabolic recombination (Romo-González et al., 2022), and signaling pathway dysregulation (Martelli et al., 2011). ALL primarily affects children, and the number of children who die from ALL is the highest number of pediatric tumor deaths yearly. Childhood ALL used to have a high mortality rate. However, disease-risk stratification and the development of intensified chemotherapy protocols greatly improved the outcome of children with ALL (Kato and Manabe, 2018). Today, the disease is highly curable in pediatric patients, with a long-term survival rate of more than 90%. It is important to improve the prognosis of children, and studies have confirmed that the prognosis of children is closely related to treatment response, and children are classified as high-risk or standard-risk based on age or genotype (Pui et al., 2011). Many factors could affect the prognosis of ALL, and they interact with each other. If these factors can be grasped, they can guide clinical treatment to a certain extent (Isakoff et al., 2013; Teachey and Pui, 2019).

The essential elements required by the human body are divided into trace elements and macronutrients according to their contents. Elements containing less than 0.01% of the human body are called trace elements. Trace elements are involved in the composition of enzymes, constitute important carriers and electron transfer systems, are also involved in the synthesis of certain hormones and vitamins, and are directly related to certain diseases. The World Health Organization has published 19 known trace elements categorized into three groups: essential, probably essential, and potentially toxic. In addition, there are 5 or more trace elements considered essential or probably essential for the physiological activity of humans and animals, including ferrum, iodine, zinc, copper, selenium, nickel, and other elements. Numerous studies have shown that trace elements are closely related to the development of tumors (Kohzadi et al., 2017; Cilliers et al., 2020; Cabral et al., 2021; Iqbal and Ali, 2022). Trace elements of the organism are a class of factors with multiple functions that are directly involved in cell proliferation, differentiation, and maturation. Their increase or decrease may cause metabolic disorders in the body and causes disease and even tumor development (World Health Organization, 1996).

ALL is a complex process involving multiple factors, genes, and stages (Pui et al., 2019), but the cause of the disease still needs to be fully understood. It is generally believed to be related to heredity, acquired gene mutations, environmental pollution, and other factors (Asai et al., 2013; Medina-Sanson et al., 2020). Many scholars have found that changes in serum trace elements are closely associated with the development of ALL. However, children’s trace elements are significantly reduced after induction chemotherapy, and to some extent, it is beneficial for children to supplement trace elements in a planned way during chemotherapy (Zekavat et al., 2021). This paper intends to review the research progress on the relationship between trace elements and ALL to provide more information on trace elements for the diagnosis, treatment, and prevention of ALL.

### The changing trend of trace elements in patients with ALL and its possible mechanism

#### Zinc and ALL

Zinc can act as a functional component or activator of many enzymes, and there are 18 known zinc enzymes and 14 other enzymes that require zinc to be activated. Among these DNA and RNA polymerases are protein and nucleic acid synthesis enzymes. These enzymes are widely present in human metabolism, mainly in sugar, protein, fat, and nucleic acid metabolism. Research revealed that intracellular zinc maintains homeostasis. Not only zinc deficiency but also zinc overload can be cytotoxic. The homeostasis of zinc can be attributed to the zinc-binding property of metallothioneins (MT) (Jarosz et al., 2017). Studies show that zinc supplementation can increase MT mRNA abundance (Aydemir et al., 2006), protect DNA and reduce reactive oxygen species (ROS) accumulation (Jing et al., 2016). On the contrary, zinc deficiency can inhibit MT expression and further induce ROS content increase (Kang et al., 2015), and there is a close relationship between oxidative stress and ALL, which may be one of the possible relationships between zinc and ALL.

Zinc deficiency can lead to diminished immune function, resulting in atrophy of immune organs and decreased immunity. Many malignant tumors, including liver, stomach, colon, lung, Hodgkin’s lymphoma, leukemia, and kidney cancer, have significantly related to lower zinc levels.

Zinc deficiency is common in ALL patients. The serum zinc concentration was initially low for leukemia patients undergoing allogeneic bone marrow transplantation. In addition, zinc can also reduce the severity and duration of oral mucositis after anti-tumor treatment for ALL. Zinc was even assessed as a marker for predicting response to chemotherapy. The possible reasons for the decrease of zinc content in ALL patients include: the strong metabolism of leukemia cells, which significantly increases zinc consumption in the body; The tumor induces the accumulation or excretion of zinc in the liver and other tissues and organs to increase, leading to the decrease of zinc in the blood; Some oncogene expression products can inhibit the synthesis of zinc absorption proteins, thus directly leading to the reduction of zinc levels in leukemia patients. Related research evaluated the possible correlation between trace elements and ALL, and elements such as zinc were even introduced as adjuvant therapy (Gutiérrez-Vargas et al., 2020).

#### Copper and ALL

Copper is an important component of more than 30 copper-containing enzymes and biologically active proteins in the body, and most of the copper-containing enzymes belong to the oxidative enzyme class, which are involved in the metabolism of catecholamine hormones and melanin production, and have important effects on the function of the central nervous system, intelligence, and mental status, the function of the immune system, and the function of the endocrine system. It has been suggested that excess copper may increase the free radical content and aggravate the occurrence of diseases such as aging and malignancy. Elemental copper acts as a tumor angiogenic factor, which causes tissue malignancy or accelerates tumor growth by competing for binding microsites on cells and altering the activity of some enzymes.

In the serum of patients with ALL, it has been found that copper levels are significantly increased (Valadbeigi et al., 2019). Studies have revealed that the serum copper level (SCL) of cancer patients is high, and its possible mechanism is that the increase of sialidotransferase on the surface of tumor cells leads to the reduction of ceruloplasmin decomposition and the increase of copper load in the body (Fisher and Shifrine, 1978).

Copper forms coordination compounds in liver cells with proteins, amino acids, and other substances. These compounds are highly liposoluble and stable in the body and easily interact with enzymes, nucleic acids, and other macromolecules, leading to malignant differentiation and proliferation of cells. As mentioned above, the dysregulation of signaling will lead to ALL, Martelli et al. revealed that target inhibition of PI3K or mTOR pathway might be a potential therapy (Martelli et al., 2011), and recently Banerjee et al. used copper chelate inducing ALL apoptosis through redox imbalance and inhibition of PI3K/Akt expression (Banerjee et al., 2016), and SCL may be an evaluation of the disease (Legutko, 1978). Hence, copper overload may disturb the physiological signaling pathway and lead to leukemia, and the role of copper chelators or reducing the copper load in hematological malignancies needs further investigation (Kaiafa et al., 2012).

#### Selenium and ALL

Many experimental studies have shown that trace element selenium has a significant anticancer effect. Selenium activates lymphocytes, stimulates immunoglobulin and antibody production, and enhances the body’s immune system. The blood selenium level of cancer patients is lower than normal, and the cancer mortality rate is also negatively correlated with the blood selenium level (Zou et al., 2002).

Epidemiological studies also suggest that the blood selenium concentration of people affected by diet and geological environment negatively correlates with the incidence and mortality of malignant tumors. As a regulating element, selenium can enhance the human immune system (El-Bayoumy, 2001).

Many investigators have confirmed the significant decrease in serum selenium levels in ALL patients. Some studies have measured the whole bone marrow fluid selenium level and found that the selenium level is reduced, and hair selenium level in children with leukemia decreased significantly (Ozgen et al., 2007). However, the causal relationship between selenium level and leukemia is still unclear, and it seems that a low concentration of selenium can cause Glutathione peroxidase (GPx) dysfunction (Nogales et al., 2013), it acts as a peroxide scavenging enzyme and reduced activity leads to ROS accumulation and DNA injury (Miyamoto et al., 2003), resultantly leukemia progression (Mannan et al., 2021). A related study found that selenium over physiological doses but below toxic doses can cause apoptosis in cancer stem cells in acute leukemia by promoting JNK1 phosphorylation, and the expression of cell cycle regulators, p21 and p27, was prominently increased (Wu et al., 2014). Certain selenium-based compounds can selectively kill ALL cells by inducing cell cycle arrest and ROS-mediated apoptosis through the mitochondrial signaling pathway. In conclusion, it is clear that the serum selenium level in ALL patients decreases, and the efficacy of appropriate treatment methods may also be related to the increase in selenium level (Mikac-Dević et al., 1990; Wu et al., 2017).

#### Ferrum and ALL

The mechanisms related to cancer may be that ferrum is needed as a cofactor for cell proliferation and growth, and research revealed that ferrum deficiency reduces the aggressiveness of tumor cells (Moussa et al., 2019), which provides some clues for leukemia treatment. Ferrum can promote the production of reactive oxygen species, ferrum overload destroys the body’s immune surveillance of malignant cells, and ferrum overload antagonizes other micronutrients (Rouault and Tong, 2008; Miller et al., 2011). More and more attention has been paid to the problem of ferrum load in children with ALL (Eng and Fish, 2011). You et al. claim that ferrum overload enhances the metastasis and growth of tumor cells. They found that deferoxamine (DFO) prevents ALL proliferation and induces cell apoptosis by down-regulating the ROS/HIF-1ɑ, Wnt/β-catenin, and p38/MAPK/ERK signaling pathway (You et al., 2022). Moreover, there is a parallel relationship between the serum ferritin content and the number of immature cells (Moafi et al., 2017; Abedi et al., 2020). Red blood cell infusion is a common treatment method for ALL children receiving chemotherapy. Therefore, ferrum deposition in different organs in the body is closely related to red blood cell infusion (Unal et al., 2014).

#### Other trace elements

Although the molecular mechanism is still unclear, Nickel is still considered a carcinogenic trace element (Lu et al., 2005). It was observed that the urinary nickel content increased significantly in children with ALL (Yang et al., 2008), and Afshin et al. found that nickel can stimulate cell proliferation through NADPH oxidase/ROS-dependent mechanism, which might be accountable for stimulating the growth of cancer stem cells (Mohammadi-Bardbori and Rannug, 2014). Moreover, urinary 8-hydroxy-2′-deoxyguanosine and nickel are significantly associated with increased ALL risk (Yang et al., 2008). Although radioactive iodine 131 (^131^I) is known to cause chromosome aberration, there is no large-scale epidemiological data to prove that the incidence rate of ALL has significantly increased, except for a few sporadic reports of cases leading to ALL, it is necessary to monitor patients’ hematological indicators after ^131^I treatment (Piccirillo et al., 1999). In addition, the double loading of trace elements such as arsenic, cadmium, chromium, cobalt, manganese, and lead in dust is not related to a significant change of ALL risk (Whitehead et al., 2015). The changes of trace elements in ALL patients are mainly focused on the above types, while the rest are rarely reported.

#### Trace elements affect DNA synthesis and participate in regulating ALL

The characteristics of ALL may be related to the gene changes of differentiation and proliferation of lymphoid precursor cells (Malard and Mohty, 2020). Genomic studies have now clarified the subclassification of ALL and demonstrated the close interaction between genetic and somatic genetic changes in the biology of ALL. Many of these changes are of great significance to the diagnosis and risk stratification of ALL and the use and development of new targeted therapies (Inaba and Mullighan, 2020). As we all know, DNA is the main genetic material molecule, and its damage is a regulatory system responsible for maintaining genomic integrity and stability. Evidence suggests that leukemic cells’ pathogenesis and phenotypic characteristics result from a combination of specific targeted and genome-wide alterations of DNA methylation (Burke and Bhatla, 2014; Hale et al., 2017). In addition, defective components in DNA damage and repair mechanisms are the root cause of the development and progression of various cancers (Samavarchi Tehrani et al., 2018). Trace elements are essential to all biological systems and cellular homeostasis.

Furthermore, some metal ions play a role in maintaining genomic stability. For example, the zinc-binding structure in DNA processing proteins contributes to the fidelity of DNA repair and tumor suppression processes to a large extent. It has been confirmed that DNA damage is involved in the occurrence and development of ALL, and the effectiveness of some therapeutic schemes may be related to the expected repair of DNA damage (Dincer et al., 2015). Trace elements play an important role in the synthesis of nucleic acids, and there are changes in trace elements in leukemia patients. Changes in trace elements represented by selenium and zinc can affect DNA damage and repair mechanisms and participate in the anticancer process. The zinc content of histones in lymphocytes of leukemia patients is significantly lower than that of normal subjects, and zinc deficiency in the body can reduce the activity of DNA polymerase, thus disrupting the body’s normal cell division and maturation process. The trace element selenium is involved in the anticancer process in several ways, and selenium can effectively reduce multiple DNA damages induced by carcinogens and promote damage repair (Lawson, 1989).

#### Possible mechanism of trace elements regulating free radical level in ALL

Under normal conditions, the organism has a complete antioxidant system. In malignant diseases of the hematological system, trace element metabolism is disturbed to varying degrees, affecting the activity of antioxidants and accelerating the disease’s development. It is believed that when malignant diseases occur in the blood system, the balance between pathogenic factors, microelements, nutritional proteins, and antioxidant activities has changed, or the original balance has been disturbed, leading to further deterioration of the blood system diseases. The accumulation of ROS caused by the consumption of antioxidants can further promote gene mutation and lipid peroxidation damage. The most important physiological function of selenium is antioxidation. Selenoproteins are the main source of selenium antioxidation. One of the important components of enzymes includes the trace element selenium. GPX is an antioxidant enzyme that widely exists in tissues. Its active center is selenocysteine. GPX can eliminate ROS and protect cells from oxidative damage (Chen et al., 2000; El-Bayoumy, 2001). Glutathione S-transferase (GST) is a phase II enzyme with detoxification in tissues, which can play a role in scavenging lipid peroxides when GPX activity is low. The activity of GPX and GST can reflect the body’s anti-oxidative damage ability and oxidative stress level. The study showed that GST activity in ALL patients is significantly increased and is also an indicator of prognosis (Koberda and Hellmann, 1991; Zhang et al., 2005).

Trace elements play an important role in protecting against free radical damage and protecting normal cell structure and metabolism; oxygen radicals can cause cell mutations, and Glutathione peroxidase (GSH-Px), superoxide dismutase (SOD), and catalase are scavengers of free radicals and reactive oxygen species in the body while protecting the membrane system of cells from damage and preventing cell carcinogenesis. Meanwhile, Selenium, ferrum, copper, zinc, and manganese are these enzymes’ structural components and activity centers. Selenium has a strong anti-free radical effect, inhibits oxidative damage to the viral genome, limits viral pathogenic mutations, and selenium regulates the activity of the selenium-containing enzyme glutathione peroxidase (Wilke et al., 1992). Zinc is closely associated with carcinogenic and anticancer effects, and zinc deficiency is carcinogenic, as zinc is associated with maintaining compartment closure, preventing a free radical attack on cells, and protecting cell membranes and normal cell division.

In patients with ALL, glutathione peroxidase (GSH Px), superoxide dismutase (SOD), and catalase are scavengers of the body’s free radicals and active oxygen species. Selenium, ferrum, copper, zinc, and manganese are these enzymes’ structural components and active centers. Trace elements play an important role in preventing free radical damage and protecting normal cell structure and metabolism in patients with ALL, which is closely related to anticancer effects.

The study found that the activity of antioxidant enzymes in children with ALL was lower than in controls, and DNA base damage was worse than in controls (Sentürker et al., 1997), supporting the notion of redox imbalance. Kennedy et al. also found that the antioxidant status in children with ALL decreased from diagnosis through the first 6 months compared to control and increased after treatment (Kennedy et al., 2005). Although leukemia therapy was successful, the treatment-related toxicities still led to some fatalities. Tonbary et al. found that adding additional antioxidants (vitamin E, N-acetyl cysteine) can reduce morbidity and transfusion rates (Al-Tonbary et al., 2009), which is beneficial to the prognosis.

**FIGURE 1:**
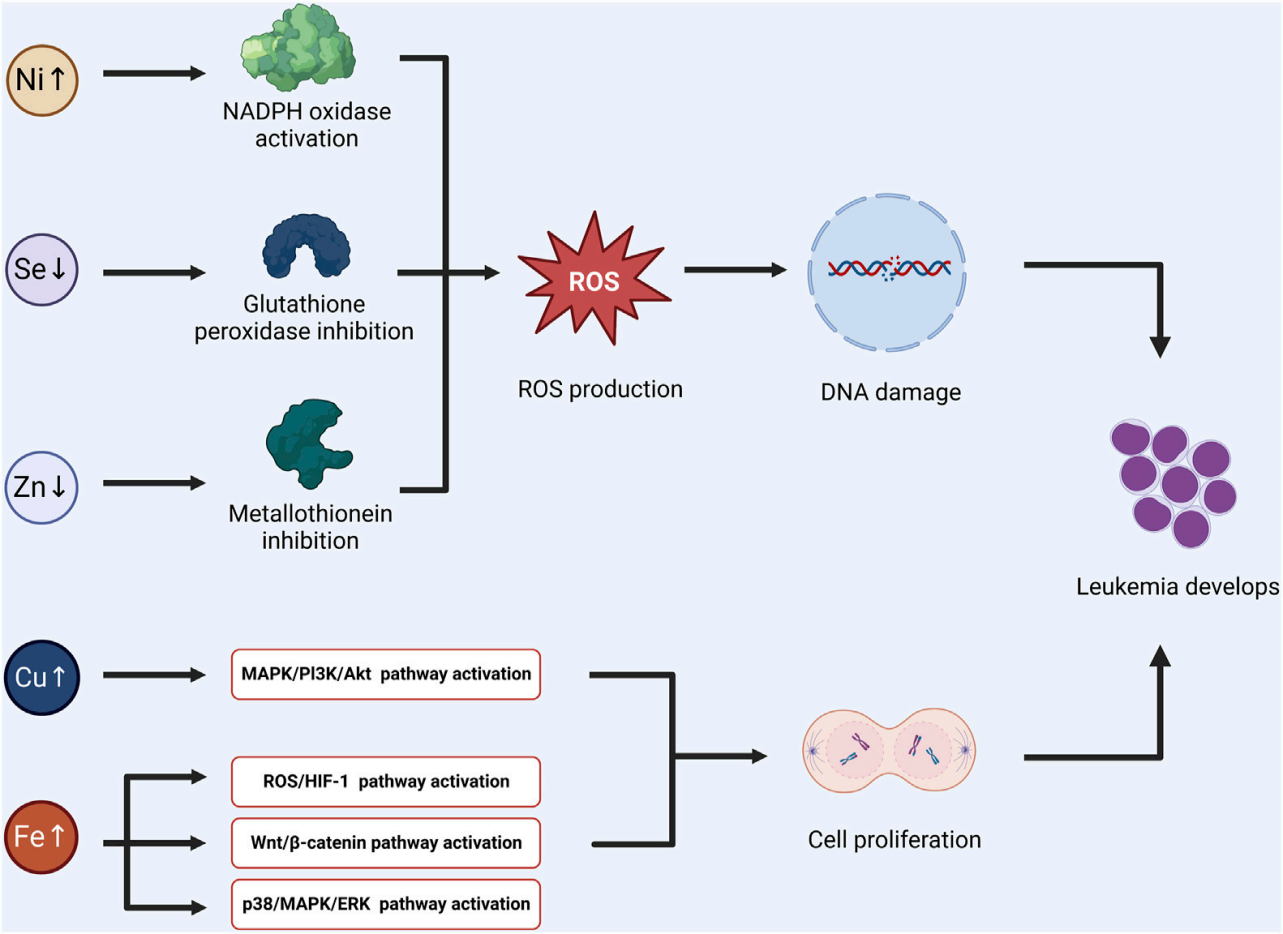
Some trace elements can affect the activity of related enzymes, induce redox imbalance, and lead to DNA damage and mutation. The other trace elements may promote the activation of signal pathways, and induce the proliferation and division of tumor stem cells. Combining the above two mechanisms, the possible pathogenesis of acute lymphoblastic leukemia induced by trace elements was summarized. (Ni: Nickel, Se: Selenium, Zn: Zinc, Cu: Cupper, Fe: Ferrum).

On the contrary, increased ROS concentration also can be an efficient system to destroy leukemia cells (Allegra et al., 2022). Therefore, further clinical big data research is needed on the need for antioxidant adjuvant therapy (Figure 1).

#### Study on the relationship between trace elements and clinical efficacy of ALL

Once in a therapeutic trial, children with acute lymphoblastic leukemia treated with chemotherapy were divided into two groups: chemotherapy plus zinc + ferrum and chemotherapy alone, and the time required to start and complete remission was significantly shorter in the chemotherapy plus zinc + ferrum group than in the chemotherapy alone group. They found that the bone marrow naive cell count decreased from 95% at the start of chemotherapy to 0% by day 14, and the blood picture returned to normal quickly and never relapsed during the treatment. At the same time, the number of infections decreased, and the child’s immune function was normal. Similar results were observed in 13 other children with ALL treated with zinc adjuvant therapy, suggesting that zinc adjuvant therapy may be important in the rapid and durable remission of pediatric ALL patients and warrants further study (Eby, 2005).

Due to the important role of selenocysteine in leukemogenesis found in studies at the cellular and animal levels, a clinical study by Weisberger et al. found (Weisberger and Suhrland, 1956) that both acute leukemia patients had a rapid decrease in total leukocyte count after oral administration of selenocysteine, along with a reduction in spleen size. Moreover, selenocysteine is equally effective in those leukemias insensitive to other chemotherapeutic agents and refractory to treatment.

In a study of sodium selenite supplementation in the population, selenium was found to have some cancer-inhibitory effects. Pazirnadhe et al. (Pazirandeh et al., 1999), in an experiment on blood selenium concentrations before and after chemotherapy in 40 children with acute lymphoblastic leukemia, found significant differences in serum selenium levels in children with leukemia before and after chemotherapy, which could be considered that the action of chemotherapeutic agents caused the decrease in serum selenium levels. *In vitro* tests have shown that selenium can effectively inhibit the growth of leukemia cells, induce the differentiation and maturation of a few leukemia cells, reduce the toxicity of some chemotherapeutic drugs, and promote the application of selenium compounds in the clinical treatment of leukemia. Jiang et al. observed that the proliferation process of leukemic cell line HL-60 was significantly inhibited by sodium selenite in an *in vitro* cell culture experimental study.

In clinical aspects, ALL patients with appropriate amounts of selenium can improve the function of the patient’s immune system and enhance the body’s anticancer and anticancer effects. In addition, several studies have confirmed that selenium can enhance the sensitivity of tumors to other chemotherapeutic drugs. Therefore, selenium plays an important role in tumor prevention, treatment, and prognosis evaluation (Figure 1).

## Summary and prospects

Trace elements are indispensable for maintaining the normal function of the body. Under normal physiological conditions, the oxidative and antioxidant systems of the body are in balance. The occurrence of leukemia may cause disorders in the metabolism of trace elements, resulting in an imbalance between the oxidative and antioxidant systems and accelerating the development of the disease. The imbalance of trace elements in the body causes impaired synthesis of ribonucleic acid and histones, which affects cell proliferation and differentiation and leads to the development of ALL. In-depth research and elucidation of the relationship between trace elements and acute lymphoblastic leukemia will help to deepen the understanding of the causes of acute lymphoblastic leukemia and improve the treatment methods, as well as contribute to the prevention of the disease. There are no randomized controlled studies on trace elements in humans with ALL in a large sample, and no relevant literature support antioxidants and micronutrient supplementation as adjuvant therapy. In this review, we selected some specific trace elements to explore their possible contribution to ALL and tried to find possible prognostic indicators and preventive measures to provide more ideas for clinical practice.
